# Comparison of Micro-Mixing in Time Pulsed Newtonian Fluid and Viscoelastic Fluid

**DOI:** 10.3390/mi10040262

**Published:** 2019-04-18

**Authors:** Meng Zhang, Wu Zhang, Zhengwei Wu, Yinan Shen, Yicheng Chen, Chaofeng Lan, Fengchen Li, Weihua Cai

**Affiliations:** 1 School of Energy Science and Engineering, Harbin Institute of Technology, Harbin 150001, China; cychit@163.com (Y.C.); lanchaofengl@126.com (C.L.); lifch@hit.edu.cn (F.L.); 2 School of Engineering and Applied Sciences, Harvard University, Cambridge, MA 02138, USA; zh0002wu@outook.com (W.Z.); yinanshen@g.harvard.edu (Y.S.); 3 School of Physics and Electronics, Guangzhou University, Guangzhou 510000, China; 4 Department of Biomedical Engineering and Biotechnology, University of Massachusetts Lowell, Lowell, MA 01854, USA; zhengwei_wu@student.uml.edu

**Keywords:** microfluidic mixing, pulsed flow, viscoelastic fluid, Newtonian fluid

## Abstract

Fluid mixing plays an essential role in many microfluidic applications. Here, we compare the mixing in time pulsing flows for both a Newtonian fluid and a viscoelastic fluid at different pulsing frequencies. In general, the mixing degree in the viscoelastic fluid is higher than that in the Newtonian fluid. Particularly, the mixing in Newtonian fluid with time pulsing is decreased when the Reynolds number *Re* is between 0.002 and 0.01, while it is enhanced when *Re* is between 0.1 and 0.2 compared with that at a constant flow rate. In the viscoelastic fluid, on the other hand, the time pulsing does not change the mixing degree when the Weissenberg number *Wi* ≤ 20, while a larger mixing degree is realized at a higher pulsing frequency when *Wi* = 50.

## 1. Introduction

In the swift development of the micro-fabrication process, microfluidic technology has been widely recognized in many practical applications such as medical diagnostics [1], biological analysis [2,3], chemical analysis [4,5,6], and heat and mass transfer [7,8], etc. In these applications, reagent mixing is usually a critical process, which largely affects the efficiency and performance of the work. Conventionally, a good and fast mixing relies on the chaotic and turbulent flow instabilities of the fluid and requires a huge Reynolds number of the flow [8,9,10,11]. However, in the microfluidic mixer, the large Reynolds number is impeded due to the micro-scaled structural dimensions, and the fluid is usually confined as laminar flow. As a result, the mixing mostly relies on the diffusion, which requires long flow distance and is very inefficient.

Various approaches have been developed to improve the microfluidic mixing, and are usually categorized into active methods and passive methods [12,13,14]. For passive methods, the microchannel configurations are optimized to increase the reagent contact area or contact time. This is usually based on the fluid lamination or the chaotic advection [15,16]. In a multi-lamination mixer, four inlets were used to reduce the thickness of the fluid layer, and a throughput of 30 μL/min was achieved [17]. Another passive mixer improved the efficiency by rotation, splitting and recombination of the fluids in the microchannel system, in which the interfacial area between two fluids was increased by many times [18]. A chaotic advection-based microfluidic mixer usually increases the efficiency by splitting, stretching, folding and breaking up the fluids [12]. Such a mixer usually requires a 3-dimensional (3D) structure. For example, a 3D mixer consisting of capillaries with different angles was developed, and the mixing efficiency was improved even with a small pressure drop [19]. A mixer with 3D V-shaped channels and triangular chambers was also designed which realized a mixing efficiency as high as 90% at *Re* less than 150 [20]. In contrast to the passive mixer, the active mixer relies on the external fields to stir the fluid and improve the mixing efficiency. Different external excitations are proposed such as acoustic waves [21], ultrasonics [22], electro-hydrodynamics [23,24] and magnetohydrodynamic pumping [25].

Besides the reliance on external fields, the flow is also controlled by introducing time pulsing to the flow [26,27,28]. Utilizing the time pulsing, less energy is consumed compared with the active method, and the mixer configuration can be simplified greatly compared with that in the passive method. For example, the structure of “├”, “Y”, and “T” shape have been reported to improve the mixing efficiency by mechanically pumping two reagents in a sinusoidal, periodic fashion [29,30]. Aperiodic perturbation flows were also studied for the improvement of mixing in the microfluidic channel [31].

Despite the fact that much research has focused on the time pulsing mixing, this study mainly concentrates on the mixing in a Newtonian fluid. In fact, the rheological properties of the fluid also have a high impact on the mixing efficiency. Recently, viscoelastic fluid is proposed to improve the mixing efficiency in the passive microfluidic channels [32,33,34].

In the viscoelastic fluid, elastic stress only decays after a characteristic time due to its elasticity. For its viscoelastic nature, the viscoelastic fluid can realize chaotic vortices even at a low Reynolds number condition [34,35]. In this paper, by applying the pulsing time approach, we experimentally compared the viscoelastic fluid mixing with the Newtonian fluid mixing in a T-junction microfluidic channel. A time pulsing with square wave modulation is applied to the flow, and the impact of the pulsing frequency and the flow rate on the mixing are investigated for both fluids.

## 2. Materials and Methods

### 2.1. Micro-Mixer Design and Fabrication

**Figure 1:**
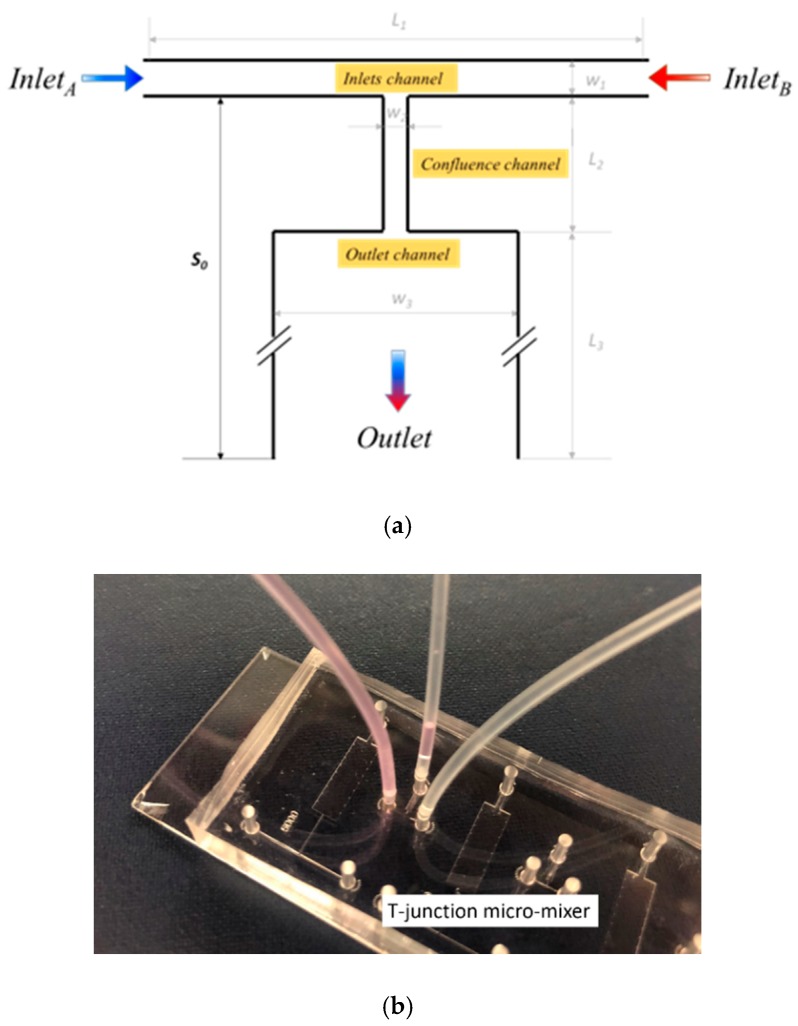
(**a**) The design and (**b**) the fabricated chip of the T-junction microfluidic mixer.

The T-junction microfluidic mixer is designed as shown in Figure 1a. Fluids of the same type are injected into two inlets *I*_A_ and *I*_B_ of the T-junction. The fluid on one side contains a small amount of fluorescent dye for experimental view. The two fluids are confluent at the center of the inlet channel and flow into a narrow channel before finally entering into an expanded mixing channel. The fluorescent color in the mixing channel is monitored to determine the mixing efficiency at the flow distance, *S*_0_, from the inlet channel. The length and width of the inlet channels are *L*_1_ = 2 mm and *W*_1_ = 100 µm, respectively. The confluence channel has dimensions of *L*_2_ = 600 µm and *W*_2_ = 50 µm, and the mixing channel has dimensions of *L*_3_ = 2 mm and *W*_3_ = 600 µm. The mixer structure has a depth of *h* = 100 µm, and the outlet is set at the end of the mixing channel.

The T-junction is fabricated using standard soft lithography processes. The mold of the design is first fabricated by spinning coat, exposure and development of a 100 µm thick SU8 photoresist on a silicon wafer. Then, the liquid polydimethylsiloxane (PDMS) consisting of pre-polymer (base) and cross-linker (curing agent) with a weight ratio of 10:1 is poured onto the SU8 mold. The PDMS becomes solid after 4 h of heating in the 65 °C oven. The solid PDMS with the mixer pattern is bonded to a glass slide after plasma treatment, and the microfluidic channel is formed. The completed T-junction mixer chip is shown in Figure 1b.

### 2.2. Materials and Underlying Physics

In the experiment, we studied both the Newtonian fluid mixing and viscoelastic fluid mixing. In the Newtonian fluid, the response of a simple shear has a linear relationship between the applied shear stress and the rate of shear, i.e., $\sigma_{yx}=\eta {\dot{\gamma }}_{yx}$, where $\sigma_{yx}$ is the shear stress, ${\dot{\gamma }}_{yx}$ is the shear rate and η is the viscosity value of the fluid. The relative balance between the inertial and viscous forces are normally characterized with the dimensionless Reynolds number, defined as Re=ρUL/η, where L is the characteristic length of the channel, U is the flow rate in the channel and ρ is the density of the fluid. In the microfluidic channel, the Re number is usually smaller than 100, and often smaller than 1 due to the reduced length scale of the channel. The viscous force is dominant to the inertial force and the fluid is usually laminar. So the mixing only depends on the molecular diffusion [36].

The viscoelastic fluid usually contains polymer chains, which can be stretched by the stress from the fluid and relax to an equilibrium state when the stress is released. Therefore, significant elasticity is manifested, and the flow behaviors are enriched. The elasticity can be characterized using the Weissenberg number, defined as Wi=λU/L, where λ is the relaxation time, referring to the characteristic stretch-relax time of the polymer. Wi qualifies the nonlinear response of the fluid. The onset of elastic instabilities at high Wi is a hallmark of viscoelastic fluid, even under creeping flows [37]. Such instabilities were experimentally observed in a number of flow geometries, such as Taylor–Couette, contraction and lid-driven cavity flows, etc [38,39,40]. Currently, it is widely accepted that the instability is related to streamline curvature, which generates tensions along streamlines, leading to flow destabilization [36].

Here, the expanded T-junction mixer introduces the stress to the polymer in the viscoelastic fluid. For the mixing comparison, the Newtonian fluid, 75% glycerol is used, which has a viscosity of 25 mPa·s. For the viscoelastic fluid, 100 ppm polyacrylamide (PAM) with a molecular weight of 18 million is used, which has a similar viscosity (27 mPa·s) with that of glycerol at the shear rate of 100 1/s and relaxation time *λ* of about 0.12 s.

### 2.3. Experimental Setup

In the mixing analysis, the same types of fluids are injected from the two inlets of the T-junction mixer, which are named fluid A and fluid B, respectively. Rhodamine B is diluted in fluid A as fluorescent dye with a concentration of 10 µg/mL. The dye is excited by a 475 nm laser, and the fluorescent color in the channel is recorded as the grey value *C*_i_ at every pixel *i*. The grey value depends on the dye concentration and can be used to derive the mixing efficiency. The flow rate Q is modulated by a time pulsing signal with a square wave function at I_A_ while at I_B_, it is a constant. The flow rates are expressed as:

(1a) $$Q_{1}\left(t\right)=Q_{0}\left\{1+sgn\left[sin\left(2\pi ft\right)\right]\right\}$$

(1b) $$Q_{2}=Q_{0}$$

where $Q_{0}$ is the flow rate amplitude and *f* is the time pulsing frequency. The flow rate at I_A_ oscillates between 0 and 2$Q_{0}$ with a period of 1/*f* and a duty cycle of 50%. The time pulsing signal will disturb the flow and further affect the mixing between fluid A and fluid B.

### 2.4. Mixing Degree Characterization

As the injected flow rate is time-dependent, both the instantaneous mixing degree $MD_{t}$ and timely averaged mixing degree $\bar{MD}$ are characterized. The $MD_{t}$ for the cross line with a distance *S*_0_ from the inlet channel at a specific recorded time *t_j_* is expressed as:

(2) $$MD_{t}=1-\sqrt{\overset{P}{\underset{i=1}{\sum }}\frac{{\left(\frac{C_{i}}{C_{0}}-1\right)}^{2}}{P}}$$

where *C_i_* is the recorded grey value at the pixel *i* on the cross line of the channel with the total pixel number of *P*. *C*_0_ is the mean value of the recorded grey values within the mixing channel. It can be seen that $MD_{t}=1$ for the full mixing case while $MD_{t}=0$ for the no mixing case. $MD_{t}$ presents the instantaneous mixing degree. To derive the average mixing degree $\bar{MD}$, we consider the fluid flowing through the cross line *S*_0_ in an adequately long recorded time range t_0_ as a collection of the fluid flowing through *S*_0_ at every recorded time *t*_i_. Therefore, the averaged mixing degree can be expressed as:

(3) $$\bar{MD}=1-\sqrt{\overset{N}{\underset{j=1}{\sum }}\frac{{\left(MD_{t=t_{j}}-1\right)}^{2}}{N}}$$

where *N* is the number of recorded frames for the concentration profile. We can also see that $\bar{MD}=1$ when the fluid is fully mixed at every instantaneous time, while $\bar{MD}=0$ when there is no mix at every instantaneous time.

## 3. Results and Discussion

### 3.1. Mixing in the Newtonian Fluid

**Figure 2:**
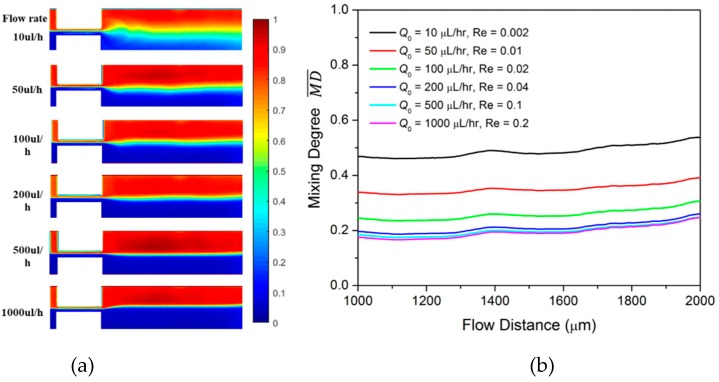
(**a**) Concentration profile of Newtonian fluid at different constant flow rates; (**b**) the averaged mixing degree $\bar{MD}$ at different constant flow rate mixing.

We first investigate the mixing in the T-junction mixer when glycerol is injected into both inlets at the same constant flow rate. The fluorescent concentration profile in the mixing channel is illustrated in Figure 2a as $Q_{0}$ varies from 10 µL/h to 1000 µL/h. The corresponding Reynolds number *Re* ranges from 0.002 to 0.2. The color in the flow map indicates the normalized concentration profile of the fluorescent dye. The red color presents a fluorescent dye concentration close to 1, while the blue color presents a concentration close to 0. At $Q_{0}$ = 10 µL/h, there is a wide green regime at the center of the channel, indicating good mixing between fluid A and the fluid B. As the flow rate increases to 50 µL/h and to 100 µL/h, the green regime largely shrinks and the mixing degree is lowered. We quantitatively characterize the mixing using the time-averaged mixing degree $\bar{MD}$ as expressed in Equation (3). $\bar{MD}$ is derived for the line across the mixing channel with a distance *S*_0_ from the inlet channel. Here, we derive the $\bar{MD}$ starting at distance *S*_1_ = 1000 µm and ending at a distance *S*_2_ = 2000 µm from the inlet channel, as shown in Figure 2b. As $Q_{0}$ increases from 10 µL/h (black line) to 50 µL/h (red line) and to 100 µL/h (green line), $\bar{MD}$ is greatly decreased in the observed area. The significant mixing decrease in the low flow rate regime stems from the diffusion time decrease when the flow rate increases. However, as the flow rate changes from 200 µL/h to 1000 µL/h, the decrease in $\bar{MD}$ is quite small. This indicates that the impact of the intrinsic diffusion on the mixing becomes weak in the high flow rate regime.

**Figure 3:**
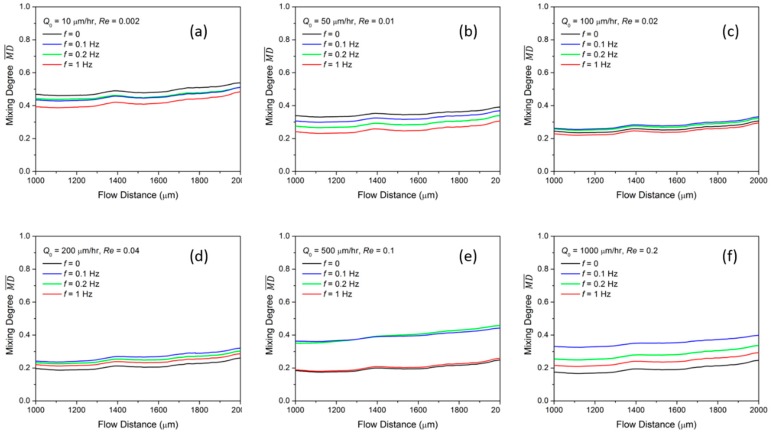
Averaged mixing degree $\bar{MD}$ of time pulsing Newtonian flow with different pulsing frequency at flow rates of (**a**) $Q_{0}$ = 10 µL/h; (**b**) $Q_{0}$ = 50 µL/h; (**c**) $Q_{0}$ = 100 µL/h; (**d**) $Q_{0}$ = 200 µL/h; (**e**) $Q_{0}$ = 500 µL/h and (**f**) $Q_{0}$ = 1000 µL/h.

Then, we modulate fluid A at a time pulsing frequency *f* = 0.1 Hz, 0.2 Hz, and 1 Hz as expressed in Equation (1a) while keeping a constant flow rate of $Q_{0}$ for fluid B. It is worth noting that the flow rate remains constant when *f* = 0. The time-averaged mixing degree $\bar{MD}$ from *S*_1_ to *S*_2_ is plotted in Figure 3a–f for different flow rates $Q_{0}$. When the fluid is at the low flow rate of 10 µL/h, $\bar{MD}$ is highest at *f* = 0 and gradually decreases as *f* increases to 1 Hz. A similar trend is also observed for the case when $Q_{0}$ = 50 µL/h. As discussed previously, the mixing largely relies on the diffusion mechanism in the low flow rate regime; we conclude that the diffusion in the microfluidic channel is affected when the disturbing frequency increases at this low flow rate.

In a higher flow rate of $Q_{0}$= 100 µL/h, the $\bar{MD}$ is generally lower compared to that at $Q_{0}$ = 50 µL/h. However, it remains almost the same when the flow rate increases from $Q_{0}$ = 100 µL/h to $Q_{0}$ = 200 µL/h, indicating a similar diffusion impact at the two flow rates. Meanwhile, when $Q_{0}$ = 200 µL/h, $\bar{MD}$ at *f* = 0 is the lowest compared to that at non-zero pulsing frequencies. This indicates that the disturbance starts to contribute to the diffusion.

As $Q_{0}$ increases from 200 µL/h to 500 µL/h, as shown in Figure 3d,e, interestingly, $\bar{MD}$ starts to increase for both *f* = 0.1 Hz and *f* = 0.2 Hz, while it remains about the same for *f* = 0 and *f* = 1 Hz. This phenomenon contradicts with the previous analysis that the mixing should be lower at a higher flow rate because of the shorter diffusion time. Here, we conclude that the impact of the intrinsic diffusion change can be neglected at this high flow rate. Instead, the disturbance contributes more significantly to the diffusion, especially at *f* = 0.1 Hz and *f* = 0.2 Hz, which makes the mixing enhanced compared to that at constant injected flow. As $Q_{0}$ increases to 1000 µL/h, the mixing pulsed by the non-zero frequency is larger compared to that at *f* = 0. It is also noticed that at a fixed flow rate, the $\bar{MD}$ is larger when a lower pulsing frequency is used. In every half period, the difference between the two fluids entering the mixing channel, noted as ΔQ, is larger for a lower *f*. Therefore, a stronger fluid oscillation in the channel can be induced.

**Figure 4:**
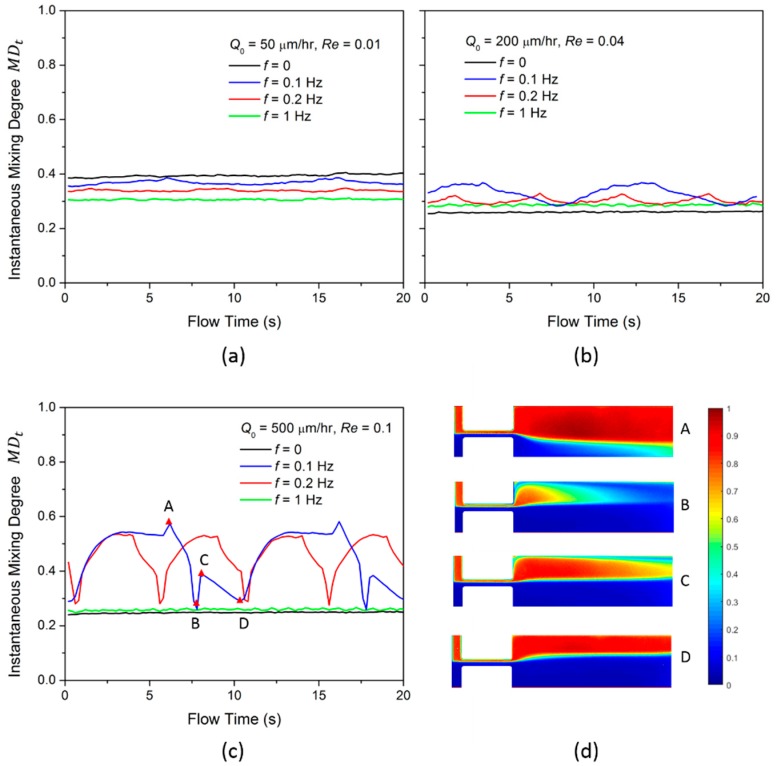
The instantaneous mixing degree $MD_{t}$ of time pulsing flow with different frequency at flow rates of (**a**) $Q_{0}$ = 50 µL/h; (**b**) $Q_{0}$ = 200 µL/h; (**c**) $Q_{0}$ = 500 µL/h at flow distance 2000 µm; (**d**) the concentration profiles at the peak-value and dip-value time instances when $Q_{0}$ = 500 µL/h.

To further understand the fluid oscillation, we analyze the instantaneous mixing degree $MD_{t}$ at the flow distance *S*_2_ = 2000 µm for 20 s, as shown in Figure 4a–c. For flow rate $Q_{0}$ = 50 µL/h, the $MD_{t}$ does not fluctuate for all disturbing frequencies because ΔQ is small at the low flow rate condition. $MD_{t}$ is highest when *f* = 0, which is due to the high intrinsic diffusion. At flow rate $Q_{0}$ = 200 µL/h, the $MD_{t}$ starts to oscillate temporally and the oscillation frequency is identical to the pulsing frequency. Meanwhile, the $MD_{t}$ for time pulsing is larger than that when *f* = 0. This confirms the contribution of diffusion disturbance to the mixing. At the high flow rate of 500 µL/h, the $MD_{t}$ oscillates significantly at *f* = 0.1 Hz and 0.2 Hz, which is much higher than that at *f* = 0.

When $Q_{0}$ = 500 µL/h and *f* = 0.1 Hz (blue line in Figure 4c), $MD_{t}$ has two peak values A and C and two dip values B and D in each period. The concentration profiles for the peak and dip values are shown in Figure 4d. At the distance *S*_2_, more red fluid oscillates across the center and mixes well with the blue fluid at the peak value A; for peak value C, more blue fluid oscillates across the center and mixes well with the red fluid. For the dip value B, the blue fluid occupies most of the channel and the mixing is low. For the dip value D, both the two fluids occupy half of the channel and little mixing occurs at the center. Therefore, we conclude the enhancement of the mixing for the Newtonian fluid can be induced by the time pulsing flow with a relatively low oscillation frequency.

### 3.2. Mixing in Viscoelastic Fluid

The viscoelastic fluid has different rheological properties compared with the Newtonian fluid as it contains elastically stretchable polymers. When injected in the microfluidic T-mixer, the fluid from two inlets will exert stress on each other and, therefore, affect the mixing. In this section, we use the 100 PAM viscoelastic fluid to investigate its mixing behavior. The same injected flow control is applied, as discussed in Section 3.1.

**Figure 5:**
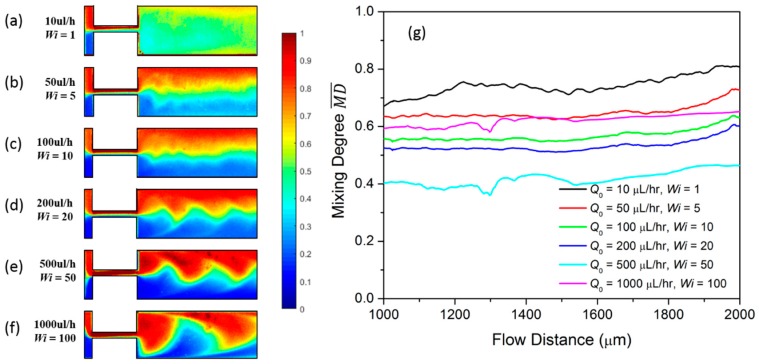
Concentration profile of viscoelastic fluid at different constant flow rates of (**a**) $Q_{0}$ = 10 µL/h; (**b**) $Q_{0}$ = 50 µL/h; (**c**) $Q_{0}$ = 100 µL/h; (**d**) $Q_{0}$ = 200 µL/h; (**e**) $Q_{0}$ = 500 µL/h and (**f**) $Q_{0}$ = 1000 µL/h; (**g**) the averaged mixing degree $\bar{MD}$ of viscoelastic fluid at different constant flow rates mixing.

The concentration profiles of the viscoelastic fluid under a constant injection flow rate in the T-mixer are plotted in Figure 5a–f. The flow rate varies from 10 µL/h to 1000 µL/h. The corresponding Weissenberg number *Wi* ranges from 1 to 100. At the low flow rate $Q_{0}$ = 10 µL/h, the green color fluid occupies most of the channel and indicates good mixing behavior. The corresponding averaged mixing degree $\bar{MD}$ as a function of the flow distance is plotted as the black line in Figure 5g. The red and the blue colored fluids appear on the two sides of the mixing channel at $Q_{0}$ = 50 µL/h, and the high mixing green regime shrinks to the center area, see Figure 5b, and $\bar{MD}$ lowers as indicated by the red line in Figure 5g. The green mixing area continues to shrink when $Q_{0}$ increases from 100 µL/h to 500 µL/h because the diffusion time is decreased for the higher flow rate. Meanwhile, it is noticed that the flow oscillates more significantly at the higher flow rate. As $Q_{0}$ increases to 1000 µL/h, the red and the blue fluids are no longer separated on two sides of the channel. Instead, they flow alternatively along the mixing channel, and one fluid pushes the other. It then reaches the channel wall on the other side and rebounds. As can be seen in the pink line in Figure 5g, this largely increases the $\bar{MD}$, which becomes 0.65 at *S*_2_ = 2000 µm.

Compared with that in glycerol, the mixing degree in PAM is significantly increased. The PAM has similar viscosity to that of glycerol. Therefore, the mixing increase must stem from the non-zero elasticity in PAM. The elasticity of the PAM arises due to the interaction between its molecular structure and the flow. The flow conditions induce a force on the polymers in the fluid, and the polymer chains are stretched and oriented. This nonequilibrium configuration imposes large anisotropic normal stresses, which, in turn, influence the flow field and the mixing effect.

**Figure 6:**
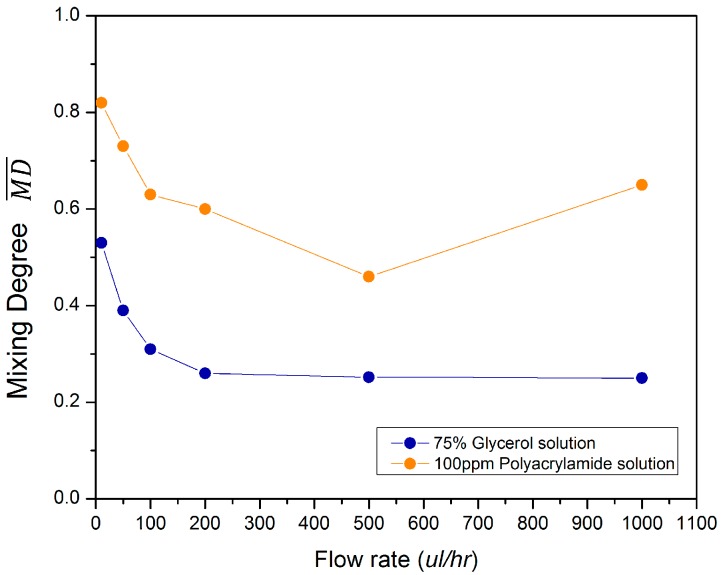
Mixing degree $\bar{MD}$ at *S*_2_ = 2000 µm at different flow rates for both glycerol and polyacrylamide (PAM).

As can be seen in Figure 2a, the concentration contour of glycerol is always symmetric along the whole channel. In addition, the concentration profile in the inlet channel and in the confluence channel are distinctly divided as red and blue colors. While for PAM, as indicated in Figure 5a–f, the concentration contour is not symmetric. In the inlet channel, there is a significant green region between the red color and blue color, indicating the mixing has already started at this point. The mixing in such a narrow area is due to the elastic stress between the two color fluids when they collide with each other. One fluid exerts an elastic force on the other in an alternating fashion, and the two fluids oscillate around the confluence point, which increases the contact area. Specifically, at the low flow rate of $Q_{0}$ = 10 µL/h, the oscillation amplitude is small and the two fluids flow side by side into the confluence channel. Due to the elastic stress at the confluence point, they continue to mix with each other for a long diffusion time. Therefore, we can observe a significant green region in the narrow confluence channel, which finally results in good mixing in the mixing channel. As indicated in Figure 6, $\bar{MD}$ at *S*_2_ = 2000 µm is 0.82. As $Q_{0}$ increases to 50 µL/h, 100 µL/h and 200 µL/h, the mixing has a similar mechanism to that when $Q_{0}$ = 10 µL/h, except that the diffusion time is decreased, thus $\bar{MD}$ decreases gradually. When $Q_{0}$ reaches 500 µL/h, the oscillation amplitude is sufficiently high so that the two fluids alternatively enter into the confluence channel; therefore, the mixing in the confluence channel is limited compared to that at a lower flow rate. This explains the large mixing decrease from 0.60 to 0.46 when $Q_{0}$ increases from 200 µL/h to 500 µL/h, respectively. As $Q_{0}$ increases from 1000 µL/h, the oscillation is so large that the fluids can reach the channel wall on the other side. As explained previously, the fluid will rebound at the wall, which increases the mixing degree.

**Figure 7:**
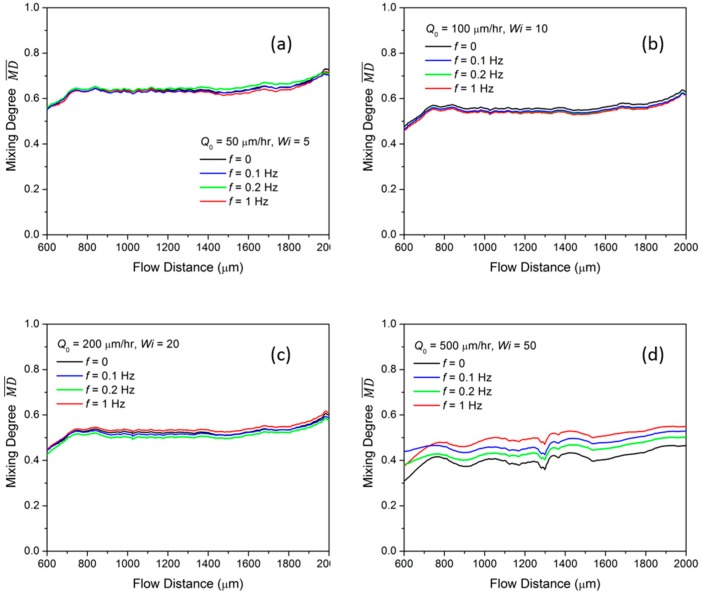
Averaged mixing degree $\bar{MD}$ of time pulsing viscoelastic flow with different frequency at flow rates of (**a**) $Q_{0}$ = 50 µL/h; (**b**) $Q_{0}$ = 100 µL/h; (**c**) $Q_{0}$ = 200 µL/h and (**d**) $Q_{0}$ = 500 µL/h.

**Figure 8:**
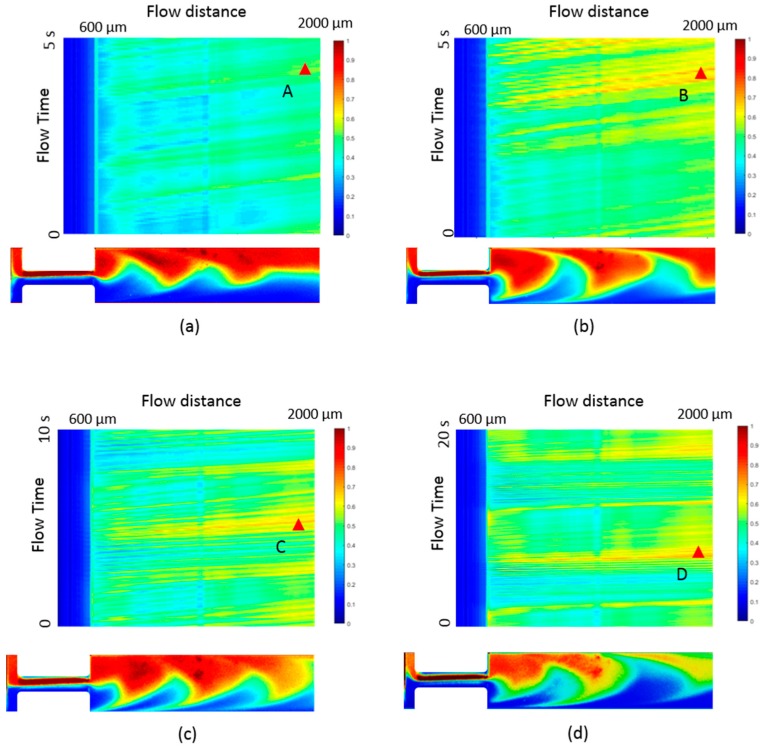
Top: The instantaneous mixing degree along the flow distance at different flow times with a flow rate of 500 µL/h at a pulsing frequency of (**a**) 0 ; (**b**) 0.1 Hz; (**c**) 0.2 Hz and (**d**) 1 Hz. Bottom: The concentration profiles at the instance of A, B, C, and D, correspondingly.

We then investigate the mixing under pulsing flow with the injection condition described in Equation (1). As shown in Figure 7a–d, the $\bar{MD}$ at flow distance *S*_1_ = 600 µm to flow distance *S*_2_ = 2000 µm in the channel is measured for different $Q_{0}$, and each sub-figure compares the $\bar{MD}$ modulated at frequency *f* = 0, 0.1 Hz, 0.2 Hz, and 1 Hz. At a fixed $Q_{0}$, lower than 200 µL/h, the $\bar{MD}$ is almost the same for different pulsing frequencies. This means the disturbance from the pulsing does not affect the mixing of the viscoelastic fluid. With $Q_{0}$ = 500 µL/h, $\bar{MD}$ is increased from 0.46 to 0.55 at *S*_2_ when the pulsing frequency increases from 0 to 1 Hz, as shown in Figure 7d. To understand the mixing improvement at the non-zero frequency condition, we investigate the instantaneous mixing degree $MD_{t}$ for a sufficient time in the mixing channel area between *S*_1_ and *S*_2_, as shown in the contour of Figure 8. The x-axis of the contour map is the flow distance and the y-axis is the flow time. It is noticed that some high mixing instances, for example, B, C and D in the contour map of Figure 8b–d, are induced during the flow and contribute to the averaged mixing. Comparably, there is no high mixing region in the constant flow condition and the flow remains almost the same at a relatively low mixing degree, like at point A in Figure 8a. The concentration profiles at points B, C and D are plotted correspondingly and compared with that at point A in the insertion in Figure 8. At point A, the red and blue fluid flow on two sides of the mixing channel and oscillate as a crescent shape due to the elastic stress. While at B, C and D, the flows have similar profiles and the red and the blue fluid flow alternatingly along the channel. This indicates that the oscillation in the constant flow condition is amplified by the time pulsing and results in a higher $\bar{MD}$. As previously discussed in Section 3.1, $\bar{MD}$ is larger at a lower frequency *f* for the mix in the Newtonian fluid. Here, in contrast, the mixing in the viscoelastic fluid, $\bar{MD}$ is larger at a higher pulsing frequency. The difference of the mixing enhancement between the Newtonian fluid and the viscoelastic fluid stems from the elastic stress in the viscoelastic fluid.

## 4. Conclusions

In conclusion: The time pulsed mixing in both the Newtonian fluid and viscoelastic fluid is experimentally investigated in a T-junction micro-mixer. The mixing efficiency of the fluid is compared for different pulsing frequencies and flow rates. In general, the mixing degree in the viscoelastic fluid is higher than that in Newtonian fluid and reaches 0.82 at *Re* = 0.002 and *Wi* = 1 in the micro-mixer. In the Newtonian fluid, compared to that at the constant flow rate, the mixing with time pulsing is decreased when *Re* is between 0.002 and 0.01, while increased when *Re* is between 0.1 and 0.2. In the viscoelastic fluid, the time pulsing does not change the mixing degree when *Wi* ≤ 20, while it realizes a larger mixing degree at a higher pulsing frequency when *Wi* = 50. The improved mixing in viscoelastic fluid stems from the strong fluid oscillation in the micro-mixer.
